# Supplementary material for: Mechanistic insight into the role of AUXIN RESISTANCE4 in trafficking of AUXIN1 and LIKE AUX1-2
Source: Plant Physiol. 2023 Sep 30;194(1):422–33. doi: 10.1093/plphys/kiad506 (PMC10756756; doi:10.1093/plphys/kiad506)
Supplement: kiad506_Supplementary_Data [file kiad506_supplementary_data.zip › Suppemental Dataset S1/5445e4f6eaa2385e-AtAXR4/hit_report.pdf]

Email	charlie.hodgman@nottinghamac.uk
Description	AtAXR4___
Date	Tue Jan 10 14:09:39 GMT 2023
Unique Job ID	5445e4f6eaa2385e

Detailed template information

#	Template	Alignment Coverage	3D Model	Confidence	% i.d.	Template Information
1	c6g75B_	Alignment	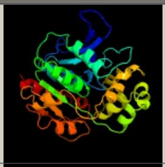	100.0	24	**PDB header:**hydrolase<br>**Chain:** B: **PDB Molecule:**common ancestor of haloalkane dehalogenase and renilla<br>**PDBTitle:** crystal structure of the common ancestor of haloalkane dehalogenases2 and renilla luciferase (anchld-rluc)<br>**PDB Entry:** PDBe RCSB PDBj
2	c2vf2A_	Alignment	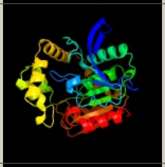	100.0	24	**PDB header:**hydrolase<br>**Chain:** A: **PDB Molecule:**2-hydroxy-6-oxo-6-phenylhexa-2,4-dienoate<br>**PDBTitle:** x-ray crystal structure of hsad from mycobacterium2 tuberculosis<br>**PDB Entry:** PDBe RCSB PDBj
3	c4mj3B_	Alignment	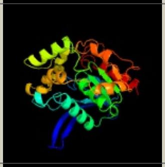	100.0	22	**PDB header:**hydrolase<br>**Chain:** B: **PDB Molecule:**haloalkane dehalogenase;<br>**PDBTitle:** haloalkane dehalogenase dmra from mycobacterium rhodesiae js60<br>**PDB Entry:** PDBe RCSB PDBj
4	d1bn7a_	Alignment	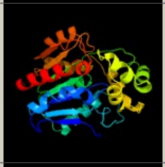	100.0	23	**Fold:** alpha/beta-Hydrolases<br>**Superfamily:** alpha/beta-Hydrolases<br>**Family:** Haloalkane dehalogenase<br>**PDB entry:** PDBe RCSB PDBj
5	c3i28A_	Alignment	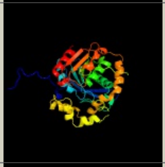	100.0	20	**PDB header:**hydrolase<br>**Chain:** A: **PDB Molecule:**epoxide hydrolase 2;<br>**PDBTitle:** crystal structure of soluble epoxide hydrolase<br>**PDB Entry:** PDBe RCSB PDBj
6	c1cr6A_	Alignment	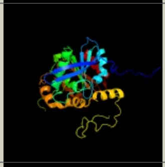	100.0	20	**PDB header:**hydrolase<br>**Chain:** A: **PDB Molecule:**epoxide hydrolase;<br>**PDBTitle:** crystal structure of murine soluble epoxide hydrolase2 complexed with cpu inhibitor<br>**PDB Entry:** PDBe RCSB PDBj
7	c6y9gB_	Alignment	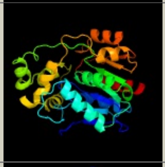	100.0	22	**PDB header:**hydrolase<br>**Chain:** B: **PDB Molecule:**ancestral haloalkane dehalogenase anchld5;<br>**PDBTitle:** crystal structure of putative ancestral haloalkane dehalogenase2 anchld5 (node 5)<br>**PDB Entry:** PDBe RCSB PDBj
8	c4ns4A_	Alignment	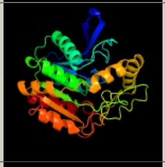	100.0	21	**PDB header:**hydrolase<br>**Chain:** A: **PDB Molecule:**alpha/beta hydrolase fold protein;<br>**PDBTitle:** crystal structure of cold-active estarase from psychrobacter2 cryohalolentis k5t<br>**PDB Entry:** PDBe RCSB PDBj
9	d1mj5a_	Alignment	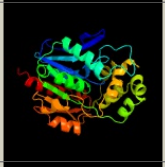	100.0	20	**Fold:** alpha/beta-Hydrolases<br>**Superfamily:** alpha/beta-Hydrolases<br>**Family:** Haloalkane dehalogenase<br>**PDB entry:** PDBe RCSB PDBj
10	c3u1tA_	Alignment	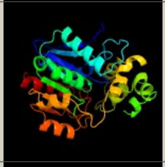	100.0	21	**PDB header:**hydrolase<br>**Chain:** A: **PDB Molecule:**dmma haloalkane dehalogenase;<br>**PDBTitle:** haloalkane dehalogenase, dmma, of marine microbial origin<br>**PDB Entry:** PDBe RCSB PDBj
11	c4c6hA_	Alignment	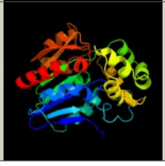	100.0	21	**PDB header:**hydrolase<br>**Chain:** A: **PDB Molecule:**haloalkane dehalogenase;<br>**PDBTitle:** haloalkane dehalogenase with 1-hexanol<br>**PDB Entry:** PDBe RCSB PDBj

12	c3a2nF_	Alignment	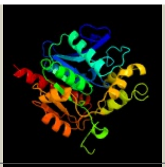	100.0	20	**PDB header:**hydrolase<br>**Chain:** F: **PDB Molecule:**haloalkane dehalogenase;<br>**PDBTitle:** crystal structure of dbja (wild type type ii p21)<br>**PDB Entry:** PDBe RCSB PDBj
13	c3wibB_	Alignment	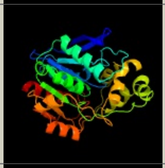	100.0	20	**PDB header:**hydrolase<br>**Chain:** B: **PDB Molecule:**haloalkane dehalogenase;<br>**PDBTitle:** crystal structure of y109w mutant haloalkane dehalogenase data from2 agrobacterium tumefaciens c58<br>**PDB Entry:** PDBe RCSB PDBj
14	c7jqzE_	Alignment	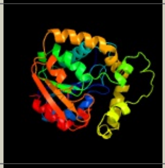	100.0	21	**PDB header:**hydrolase<br>**Chain:** E: **PDB Molecule:**alpha/beta hydrolase fold;<br>**PDBTitle:** crystal structure of cfl2 wild-type from burkholderia cenocepacia<br>**PDB Entry:** PDBe RCSB PDBj
15	d1cr6a2	Alignment	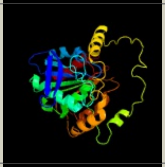	100.0	20	**Fold:** alpha/beta-Hydrolases<br>**Superfamily:** alpha/beta-Hydrolases<br>**Family:** Epoxide hydrolase<br>**PDB entry:** PDBe RCSB PDBj
16	d1zd3a2	Alignment	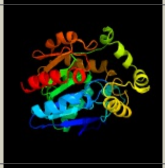	100.0	20	**Fold:** alpha/beta-Hydrolases<br>**Superfamily:** alpha/beta-Hydrolases<br>**Family:** Epoxide hydrolase<br>**PDB entry:** PDBe RCSB PDBj
17	c2e3jA_	Alignment	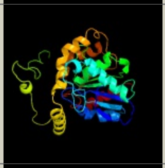	100.0	20	**PDB header:**hydrolase<br>**Chain:** A: **PDB Molecule:**epoxide hydrolase ephb;<br>**PDBTitle:** the crystal structure of epoxide hydrolase b (rv1938) from2 mycobacterium tuberculosis at 2.1 angstrom<br>**PDB Entry:** PDBe RCSB PDBj
18	c7avrG_	Alignment	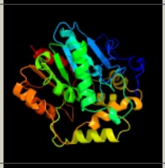	100.0	18	**PDB header:**hydrolase<br>**Chain:** G: **PDB Molecule:**haloalkane dehalogenase 1;<br>**PDBTitle:** the tetrameric structure of haloalkane dehalogenase dpaa from2 paraglaciecola agarilytica no2<br>**PDB Entry:** PDBe RCSB PDBj
19	c2qvbA_	Alignment	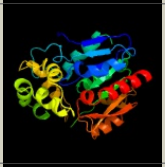	100.0	20	**PDB header:**hydrolase<br>**Chain:** A: **PDB Molecule:**haloalkane dehalogenase 3;<br>**PDBTitle:** crystal structure of haloalkane dehalogenase rv2579 from mycobacterium2 tuberculosis<br>**PDB Entry:** PDBe RCSB PDBj
20	c5xmdA_	Alignment	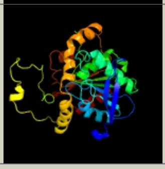	100.0	19	**PDB header:**hydrolase<br>**Chain:** A: **PDB Molecule:**epoxide hydrolase a;<br>**PDBTitle:** crystal structure of epoxide hydrolase vreh1 from vigna radiata<br>**PDB Entry:** PDBe RCSB PDBj
21	d1hkha_	Alignment	not modelled	100.0	20	**Fold:** alpha/beta-Hydrolases<br>**Superfamily:** alpha/beta-Hydrolases<br>**Family:** Haloperoxidase<br>**PDB entry:** PDBe RCSB PDBj
22	c2pseA_	Alignment	not modelled	100.0	18	**PDB header:**oxidoreductase<br>**Chain:** A: **PDB Molecule:**renilla-luciferin 2-monooxygenase;<br>**PDBTitle:** crystal structures of the luciferase and green fluorescent protein2 from renilla reniformis<br>**PDB Entry:** PDBe RCSB PDBj
23	c5ng7B_	Alignment	not modelled	100.0	20	**PDB header:**hydrolase<br>**Chain:** B: **PDB Molecule:**epoxide hydrolase;<br>**PDBTitle:** novel epoxide hydrolases belonging to the alpha/beta hydrolases2 superfamily in metagenomes from hot environments<br>**PDB Entry:** PDBe RCSB PDBj
24	d2rhwa1	Alignment	not modelled	100.0	19	**Fold:** alpha/beta-Hydrolases<br>**Superfamily:** alpha/beta-Hydrolases<br>**Family:** Carbon-carbon bond hydrolase<br>**PDB entry:** PDBe RCSB PDBj
25	c4psuA_	Alignment	not modelled	100.0	19	**PDB header:**hydrolase<br>**Chain:** A: **PDB Molecule:**alpha/beta hydrolase;<br>**PDBTitle:** crystal structure of alpha/beta hydrolase from rhodopseudomonas2 palustris cga009<br>**PDB Entry:** PDBe RCSB PDBj
26	d1a8qa_	Alignment	not modelled	100.0	20	**Fold:** alpha/beta-Hydrolases<br>**Superfamily:** alpha/beta-Hydrolases<br>**Family:** Haloperoxidase<br>**PDB entry:** PDBe RCSB PDBj
27	c6f9oA_	Alignment	not modelled	100.0	18	**PDB header:**hydrolase<br>**Chain:** A: **PDB Molecule:**haloalkane dehalogenase;<br>**PDBTitle:** crystal structure of cold-adapted haloalkane dehalogenase dpca from2 psychrobacter cryohalolentis k5<br>**PDB Entry:** PDBe RCSB PDBj

28	c2wirB_	Alignment	not modelled	98.8	20	**PDB header:**hydrolase<br>**Chain:** B: **PDB Molecule:**alpha/beta hydrolase fold-3 domain protein;<br>**PDBTitle:** hyperthermophilic esterase from the archeon pyrobaculum2 calidifontis<br>**PDB Entry:** PDBe RCSB PDBj
29	c7wwfD_	Alignment	not modelled	100.0	19	**PDB header:**biosynthetic protein<br>**Chain:** D: **PDB Molecule:**esterase;<br>**PDBTitle:** crystal structure of bioh3 from mycolicibacterium smegmatis<br>**PDB Entry:** PDBe RCSB PDBj
30	c5frdA_	Alignment	not modelled	100.0	20	**PDB header:**hydrolase<br>**Chain:** A: **PDB Molecule:**carboxylesterase (est-2);<br>**PDBTitle:** structure of a thermophilic esterase<br>**PDB Entry:** PDBe RCSB PDBj
31	c6i8wB_	Alignment	not modelled	100.0	16	**PDB header:**hydrolase<br>**Chain:** B: **PDB Molecule:**alpha/beta fold hydrolase;<br>**PDBTitle:** crystal structure of a membrane phospholipase a, a novel bacterial2 virulence factor<br>**PDB Entry:** PDBe RCSB PDBj
32	c4opmB_	Alignment	not modelled	100.0	17	**PDB header:**hydrolase<br>**Chain:** B: **PDB Molecule:**lipase;<br>**PDBTitle:** crystal structure of a putative lipase (lip1) from acinetobacter2 baumannii aye at 1.70 a resolution<br>**PDB Entry:** PDBe RCSB PDBj
33	c4inzB_	Alignment	not modelled	100.0	19	**PDB header:**hydrolase<br>**Chain:** B: **PDB Molecule:**soluble epoxide hydrolase;<br>**PDBTitle:** the crystal structure of m145a mutant of an epoxide hydrolase from2 bacillus megaterium<br>**PDB Entry:** PDBe RCSB PDBj
34	c3qitB_	Alignment	not modelled	100.0	19	**PDB header:**hydrolase<br>**Chain:** B: **PDB Molecule:**polyketide synthase;<br>**PDBTitle:** thioesterase domain from curacin biosynthetic pathway<br>**PDB Entry:** PDBe RCSB PDBj
35	c2xt0A_	Alignment	not modelled	100.0	18	**PDB header:**hydrolase<br>**Chain:** A: **PDB Molecule:**haloalkane dehalogenase;<br>**PDBTitle:** dehalogenase dppa from plesiocystis pacifica sir-i<br>**PDB Entry:** PDBe RCSB PDBj
36	c5w8pA_	Alignment	not modelled	100.0	16	**PDB header:**transferase<br>**Chain:** A: **PDB Molecule:**homoserine o-acetyltransferase;<br>**PDBTitle:** homoserine transacetylase metx from mycobacterium abscessus<br>**PDB Entry:** PDBe RCSB PDBj
37	c1u2eA_	Alignment	not modelled	100.0	18	**PDB header:**hydrolase<br>**Chain:** A: **PDB Molecule:**2-hydroxy-6-ketonona-2,4-dienedioic acid<br>**PDBTitle:** crystal structure of the c-c bond hydrolase mhpc<br>**PDB Entry:** PDBe RCSB PDBj
38	c6kxhD_	Alignment	not modelled	100.0	19	**PDB header:**hydrolase<br>**Chain:** D: **PDB Molecule:**putative hydrolase;<br>**PDBTitle:** alp1u_y247f mutant in complex with fluostatin c<br>**PDB Entry:** PDBe RCSB PDBj
39	c6unwA_	Alignment	not modelled	100.0	18	**PDB header:**hydrolase<br>**Chain:** A: **PDB Molecule:**soluble epoxide hydrolase;<br>**PDBTitle:** epoxide hydrolase from an endophytic streptomyces<br>**PDB Entry:** PDBe RCSB PDBj
40	c5xb6D_	Alignment	not modelled	99.9	18	**PDB header:**hydrolase<br>**Chain:** D: **PDB Molecule:**uncharacterized protein ycjy;<br>**PDBTitle:** crystal structure of ycjy from e. coli<br>**PDB Entry:** PDBe RCSB PDBj
41	c5cw2C_	Alignment	not modelled	100.0	18	**PDB header:**hydrolase<br>**Chain:** C: **PDB Molecule:**putative epoxide hydrolase epha;<br>**PDBTitle:** crystal structure of epoxide hydrolase a from mycobacterium2 thermoresistibile<br>**PDB Entry:** PDBe RCSB PDBj
42	d2hu7a2	Alignment	not modelled	99.6	19	**Fold:** alpha/beta-Hydrolases<br>**Superfamily:** alpha/beta-Hydrolases<br>**Family:** Acylamino-acid-releasing enzyme, C-terminal donain<br>**PDB entry:** PDBe RCSB PDBj
43	c4b9aA_	Alignment	not modelled	100.0	17	**PDB header:**hydrolase<br>**Chain:** A: **PDB Molecule:**probable epoxide hydrolase;<br>**PDBTitle:** structure of a putative epoxide hydrolase from pseudomonas2 aeruginosa.<br>**PDB Entry:** PDBe RCSB PDBj
44	c8e18A_	Alignment	not modelled	99.9	15	**PDB header:**hydrolase<br>**Chain:** A: **PDB Molecule:**secreted hydrolase;<br>**PDBTitle:** crystal structure of apo tnmk1<br>**PDB Entry:** PDBe RCSB PDBj
45	c5jrlC_	Alignment	not modelled	99.3	20	**PDB header:**hydrolase<br>**Chain:** C: **PDB Molecule:**dipeptidyl aminopeptidases/acylaminoacyl-peptidases-like<br>**PDBTitle:** crystal structure of the sphingopyxin i lasso peptide isopeptidase2 spi-isop (native)<br>**PDB Entry:** PDBe RCSB PDBj
46	c4zxiA_	Alignment	not modelled	99.4	16	**PDB header:**biosynthetic protein<br>**Chain:** A: **PDB Molecule:**tyrocidine synthetase 3;<br>**PDBTitle:** crystal structure of holo-ab3403 a four domain nonribosomal peptide2 synthetase bound to amp and glycine<br>**PDB Entry:** PDBe RCSB PDBj
47	d1brta_	Alignment	not modelled	100.0	18	**Fold:** alpha/beta-Hydrolases<br>**Superfamily:** alpha/beta-Hydrolases<br>**Family:** Haloperoxidase<br>**PDB entry:** PDBe RCSB PDBj
48	d1fj2a_	Alignment	not modelled	99.8	22	**Fold:** alpha/beta-Hydrolases<br>**Superfamily:** alpha/beta-Hydrolases<br>**Family:** Carboxylesterase/thioesterase 1<br>**PDB entry:** PDBe RCSB PDBj
49	c4uhhA_	Alignment	not modelled	100.0	17	**PDB header:**hydrolase<br>**Chain:** A: **PDB Molecule:**esterase;<br>**PDBTitle:** structural studies of a thermophilic esterase from2 thermogutta terrifontis (cacodylate complex)<br>**PDB Entry:** PDBe RCSB PDBj
50	d1qo7a_	Alignment	not modelled	100.0	18	**Fold:** alpha/beta-Hydrolases<br>**Superfamily:** alpha/beta-Hydrolases

[illegible]	[illegible]	Alignment	not modelled	[illegible]	[illegible]	**Family:** Epoxide hydrolase<br>**PDB entry:** PDBe RCSB PDBj
51	c3kdaB_	Alignment	not modelled	100.0	18	**PDB header:**hydrolase<br>**Chain:** B: **PDB Molecule:**cftr inhibitory factor (cif);<br>**PDBTitle:** crystal structure of the cftr inhibitory factor cif with the h269a2 mutation<br>**PDB Entry:** PDBe RCSB PDBj
52	c4rncB_	Alignment	not modelled	100.0	18	**PDB header:**hydrolase<br>**Chain:** B: **PDB Molecule:**esterase;<br>**PDBTitle:** crystal structure of an esterase rhest1 from rhodococcus sp. ecu1013<br>**PDB Entry:** PDBe RCSB PDBj
53	c6u2mC_	Alignment	not modelled	100.0	22	**PDB header:**hydrolase<br>**Chain:** C: **PDB Molecule:**halocamp v2;<br>**PDBTitle:** crystal structure of a halotag-based calcium indicator, halocamp v2,2 bound to jf635<br>**PDB Entry:** PDBe RCSB PDBj
54	c4d9jI_	Alignment	not modelled	100.0	16	**PDB header:**de novo protein<br>**Chain:** I: **PDB Molecule:**designed 16nm tetrahedral protein cage containing non-haem<br>**PDBTitle:** structure of a 16 nm protein cage designed by fusing symmetric2 oligomeric domains<br>**PDB Entry:** PDBe RCSB PDBj
55	c5uohA_	Alignment	not modelled	99.9	15	**PDB header:**hydrolase<br>**Chain:** A: **PDB Molecule:**carboxylesterase a;<br>**PDBTitle:** crystal structure of hip1 (rv2224c) t466a mutant<br>**PDB Entry:** PDBe RCSB PDBj
56	d1f0na_	Alignment	not modelled	99.5	18	**Fold:** alpha/beta-Hydrolases<br>**Superfamily:** alpha/beta-Hydrolases<br>**Family:** Mycobacterial antigens<br>**PDB entry:** PDBe RCSB PDBj
57	c6aaeA_	Alignment	not modelled	98.9	19	**PDB header:**hydrolase<br>**Chain:** A: **PDB Molecule:**esterase;<br>**PDBTitle:** crystal structure of chloramphenicol-metabolizaing enzyme estdl136<br>**PDB Entry:** PDBe RCSB PDBj
58	c5hk8A_	Alignment	not modelled	100.0	19	**PDB header:**hydrolase<br>**Chain:** A: **PDB Molecule:**probable pheophorbidase;<br>**PDBTitle:** crystal structure of a methylesterase protein mes16 from arabidopsis<br>**PDB Entry:** PDBe RCSB PDBj
59	c6ojdB_	Alignment	not modelled	100.0	20	**PDB header:**hydrolase<br>**Chain:** B: **PDB Molecule:**nocb;<br>**PDBTitle:** a high-resolution crystal structure of covalent complex of nocb2 thioesterase domain with fluorophosphonate nocardicin g analog<br>**PDB Entry:** PDBe RCSB PDBj
60	c4lxgA_	Alignment	not modelled	100.0	17	**PDB header:**hydrolase<br>**Chain:** A: **PDB Molecule:**mcp hydrolase;<br>**PDBTitle:** crystal structure of dxnb2, a carbon - carbon bond hydrolase from2 sphingomonas wittichii rw1<br>**PDB Entry:** PDBe RCSB PDBj
61	c5yhpB_	Alignment	not modelled	100.0	17	**PDB header:**hydrolase<br>**Chain:** B: **PDB Molecule:**cold active proline iminopeptidase;<br>**PDBTitle:** proline iminopeptidase from psychrophilic yeast glaciozyma antarctica<br>**PDB Entry:** PDBe RCSB PDBj
62	c3u0vA_	Alignment	not modelled	99.6	21	**PDB header:**hydrolase<br>**Chain:** A: **PDB Molecule:**lysophospholipase-like protein 1;<br>**PDBTitle:** crystal structure analysis of human lyplal1<br>**PDB Entry:** PDBe RCSB PDBj
63	d1b6ga_	Alignment	not modelled	100.0	15	**Fold:** alpha/beta-Hydrolases<br>**Superfamily:** alpha/beta-Hydrolases<br>**Family:** Haloalkane dehalogenase<br>**PDB entry:** PDBe RCSB PDBj
64	c1y37A_	Alignment	not modelled	100.0	16	**PDB header:**hydrolase<br>**Chain:** A: **PDB Molecule:**fluoroacetate dehalogenase;<br>**PDBTitle:** structure of fluoroacetate dehalogenase from burkholderia sp. fa1<br>**PDB Entry:** PDBe RCSB PDBj
65	c7dq9A_	Alignment	not modelled	100.0	17	**PDB header:**hydrolase<br>**Chain:** A: **PDB Molecule:**predicted hydrolases or acyltransferases (alpha/beta<br>**PDBTitle:** crystal structure of a type-a feruloyl esterase from gut alistipes2 shahii<br>**PDB Entry:** PDBe RCSB PDBj
66	d1ivya_	Alignment	not modelled	99.0	17	**Fold:** alpha/beta-Hydrolases<br>**Superfamily:** alpha/beta-Hydrolases<br>**Family:** Serine carboxypeptidase-like<br>**PDB entry:** PDBe RCSB PDBj
67	c2zyiB_	Alignment	not modelled	99.8	19	**PDB header:**hydrolase<br>**Chain:** B: **PDB Molecule:**lipase, putative;<br>**PDBTitle:** a. fulgidus lipase with fatty acid fragment and calcium<br>**PDB Entry:** PDBe RCSB PDBj
68	c5mxpB_	Alignment	not modelled	100.0	17	**PDB header:**hydrolase<br>**Chain:** B: **PDB Molecule:**alpha/beta hydrolase;<br>**PDBTitle:** haloalkane dehalogenase dmxa from marinobacter sp. elb17 possessing a2 unique catalytic residue<br>**PDB Entry:** PDBe RCSB PDBj
69	c3r3xA_	Alignment	not modelled	100.0	16	**PDB header:**hydrolase<br>**Chain:** A: **PDB Molecule:**fluoroacetate dehalogenase;<br>**PDBTitle:** crystal structure of the fluoroacetate dehalogenase rpa1163 -2 asp110asn/bromoacetate<br>**PDB Entry:** PDBe RCSB PDBj
70	c2hdwB_	Alignment	not modelled	99.9	16	**PDB header:**hydrolase<br>**Chain:** B: **PDB Molecule:**hypothetical protein pa2218;<br>**PDBTitle:** crystal structure of hypothetical protein pa2218 from pseudomonas2 aeruginosa<br>**PDB Entry:** PDBe RCSB PDBj
71	d1wpxa1	Alignment	not modelled	98.8	15	**Fold:** alpha/beta-Hydrolases<br>**Superfamily:** alpha/beta-Hydrolases<br>**Family:** Serine carboxypeptidase-like<br>**PDB entry:** PDBe RCSB PDBj

72	c7c4dA_	Alignment	not modelled	100.0	17	**PDB header:**hydrolase<br>**Chain:** A: **PDB Molecule:**putative esterase;<br>**PDBTitle:** marine microorganism esterase<br>**PDB Entry:** PDBe RCSB PDBj
73	c2y6vB_	Alignment	not modelled	100.0	15	**PDB header:**hydrolase<br>**Chain:** B: **PDB Molecule:**peroxisomal membrane protein lpx1;<br>**PDBTitle:** peroxisomal alpha-beta-hydrolase lpx1 (yor084w) from saccharomyces2 cerevisiae (crystal form i)<br>**PDB Entry:** PDBe RCSB PDBj
74	c3i1iA_	Alignment	not modelled	100.0	14	**PDB header:**transferase<br>**Chain:** A: **PDB Molecule:**homoserine o-acetyltransferase;<br>**PDBTitle:** x-ray crystal structure of homoserine o-acetyltransferase from2 bacillus anthracis<br>**PDB Entry:** PDBe RCSB PDBj
75	d1uk8a_	Alignment	not modelled	100.0	16	**Fold:** alpha/beta-Hydrolases<br>**Superfamily:** alpha/beta-Hydrolases<br>**Family:** Carbon-carbon bond hydrolase<br>**PDB entry:** PDBe RCSB PDBj
76	d1e89a_	Alignment	not modelled	100.0	17	**Fold:** alpha/beta-Hydrolases<br>**Superfamily:** alpha/beta-Hydrolases<br>**Family:** Hydroxynitrile lyase-like<br>**PDB entry:** PDBe RCSB PDBj
77	c5nfqA_	Alignment	not modelled	100.0	16	**PDB header:**hydrolase<br>**Chain:** A: **PDB Molecule:**epoxide hydrolase belonging to alpha/beta hydrolase<br>**PDBTitle:** novel epoxide hydrolases belonging to the alpha/beta hydrolases2 superfamily in metagenomes from hot environments<br>**PDB Entry:** PDBe RCSB PDBj
78	c3kxpD_	Alignment	not modelled	100.0	16	**PDB header:**hydrolase<br>**Chain:** D: **PDB Molecule:**alpha-(n-acetylaminomethylene)succinic acid hydrolase;<br>**PDBTitle:** crystal structure of e-2-(acetamidomethylene)succinate hydrolase<br>**PDB Entry:** PDBe RCSB PDBj
79	c6khlB_	Alignment	not modelled	100.0	16	**PDB header:**hydrolase<br>**Chain:** B: **PDB Molecule:**hydrolase, alpha/beta domain protein;<br>**PDBTitle:** lipase (blocked form)<br>**PDB Entry:** PDBe RCSB PDBj
80	c1mo2A_	Alignment	not modelled	100.0	20	**PDB header:**transferase<br>**Chain:** A: **PDB Molecule:**erythronolide synthase, modules 5 and 6;<br>**PDBTitle:** thioesterase domain from 6-deoxyerythronolide synthase (debs te), ph2 8.5<br>**PDB Entry:** PDBe RCSB PDBj
81	d1mo2a_	Alignment	not modelled	100.0	20	**Fold:** alpha/beta-Hydrolases<br>**Superfamily:** alpha/beta-Hydrolases<br>**Family:** Thioesterase domain of polypeptide, polyketide and fatty acid synthases<br>**PDB entry:** PDBe RCSB PDBj
82	c2o2gA_	Alignment	not modelled	99.8	21	**PDB header:**hydrolase<br>**Chain:** A: **PDB Molecule:**dienelactone hydrolase;<br>**PDBTitle:** crystal structure of dienelactone hydrolase (yp_324580.1) from2 anabaena variabilis atcc 29413 at 1.92 a resolution<br>**PDB Entry:** PDBe RCSB PDBj
83	c7wwhB_	Alignment	not modelled	99.9	18	**PDB header:**hydrolase<br>**Chain:** B: **PDB Molecule:**alpha/beta hydrolase;<br>**PDBTitle:** crystal structure of the geobacillus thermoglucosidasius feruloyl2 esterase gthfae<br>**PDB Entry:** PDBe RCSB PDBj
84	c3wwoA_	Alignment	not modelled	100.0	17	**PDB header:**lyase<br>**Chain:** A: **PDB Molecule:**(s)-hydroxynitrile lyase;<br>**PDBTitle:** s-selective hydroxynitrile lyase from baliospermum montanum (apo1)<br>**PDB Entry:** PDBe RCSB PDBj
85	c2hu7A_	Alignment	not modelled	99.9	17	**PDB header:**hydrolase<br>**Chain:** A: **PDB Molecule:**acylamino-acid-releasing enzyme;<br>**PDBTitle:** binding of inhibitors by acylaminoacyl peptidase<br>**PDB Entry:** PDBe RCSB PDBj
86	d1q0ra_	Alignment	not modelled	100.0	16	**Fold:** alpha/beta-Hydrolases<br>**Superfamily:** alpha/beta-Hydrolases<br>**Family:** Aclacinomycin methylesterase RdmC<br>**PDB entry:** PDBe RCSB PDBj
87	d1hlga_	Alignment	not modelled	100.0	14	**Fold:** alpha/beta-Hydrolases<br>**Superfamily:** alpha/beta-Hydrolases<br>**Family:** Gastric lipase<br>**PDB entry:** PDBe RCSB PDBj
88	c7qjmA_	Alignment	not modelled	100.0	15	**PDB header:**hydrolase<br>**Chain:** A: **PDB Molecule:**alpha/beta-hydrolase (202);<br>**PDBTitle:** crystal structure of an alpha/beta-hydrolase enzyme from chloroflexus2 sp. ms-g (202)<br>**PDB Entry:** PDBe RCSB PDBj
89	c5esrA_	Alignment	not modelled	100.0	15	**PDB header:**hydrolase<br>**Chain:** A: **PDB Molecule:**haloalkane dehalogenase;<br>**PDBTitle:** crystal structure of haloalkane dehalogenase (dcca) from caulobacter2 crescentus<br>**PDB Entry:** PDBe RCSB PDBj
90	d1j1ia_	Alignment	not modelled	100.0	17	**Fold:** alpha/beta-Hydrolases<br>**Superfamily:** alpha/beta-Hydrolases<br>**Family:** Carbon-carbon bond hydrolase<br>**PDB entry:** PDBe RCSB PDBj
91	c1j1iA_	Alignment	not modelled	100.0	17	**PDB header:**hydrolase<br>**Chain:** A: **PDB Molecule:**meta cleavage compound hydrolase;<br>**PDBTitle:** crystal structure of a his-tagged serine hydrolase involved2 in the carbazole degradation (carc enzyme)<br>**PDB Entry:** PDBe RCSB PDBj
92	c2vsqA_	Alignment	not modelled	99.0	14	**PDB header:**ligase<br>**Chain:** A: **PDB Molecule:**surfactin synthetase subunit 3;<br>**PDBTitle:** structure of surfactin a synthetase c (srfa-c), a nonribosomal peptide2 synthetase termination module<br>**PDB Entry:** PDBe RCSB PDBj
93	c6ix4A_	Alignment	not modelled	100.0	16	**PDB header:**hydrolase<br>**Chain:** A: **PDB Molecule:**microsomal epoxide hydrolase;<br>**PDBTitle:** structure of an epoxide hydrolase from aspergillus usamii e001 (aueh2)2 at 1.51 angstroms resolution

						**PDB Entry:** PDBe RCSB PDBj
94	c2qmqA_	Alignment	not modelled	100.0	16	**PDB header:**signaling protein<br>**Chain:** A: **PDB Molecule:**protein ndrg2;<br>**PDBTitle:** crystal structure of a n-myc downstream regulated 2 protein (ndrg2,2 syld, ndr2, ai182517, au040374) from mus musculus at 1.70 a3 resolution<br>**PDB Entry:** PDBe RCSB PDBj
95	d1a88a_	Alignment	not modelled	100.0	16	**Fold:** alpha/beta-Hydrolases<br>**Superfamily:** alpha/beta-Hydrolases<br>**Family:** Haloperoxidase<br>**PDB entry:** PDBe RCSB PDBj
96	d2h7xa1	Alignment	not modelled	100.0	19	**Fold:** alpha/beta-Hydrolases<br>**Superfamily:** alpha/beta-Hydrolases<br>**Family:** Thioesterase domain of polypeptide, polyketide and fatty acid synthases<br>**PDB entry:** PDBe RCSB PDBj
97	c4qlaA_	Alignment	not modelled	100.0	16	**PDB header:**hydrolase<br>**Chain:** A: **PDB Molecule:**juvenile hormone epoxide hydrolase;<br>**PDBTitle:** crystal structure of juvenile hormone epoxide hydrolase from the2 silkworm bombyx mori<br>**PDB Entry:** PDBe RCSB PDBj
98	c6ra2E_	Alignment	not modelled	100.0	16	**PDB header:**hydrolase<br>**Chain:** E: **PDB Molecule:**putative dioxygenase (1h-3-hydroxy-4-oxoquinaldine 2,4-<br>**PDBTitle:** structural basis for recognition and ring-cleavage of the pseudomonas2 quinolone signal (pqs) by aqdc<br>**PDB Entry:** PDBe RCSB PDBj
99	c7jqxD_	Alignment	not modelled	100.0	15	**PDB header:**hydrolase<br>**Chain:** D: **PDB Molecule:**cif-like 1 wild-type;<br>**PDBTitle:** crystal structure of cfl1 wild-type from burkholderia cenocepacia<br>**PDB Entry:** PDBe RCSB PDBj
100	c4i3fA_	Alignment	not modelled	100.0	15	**PDB header:**hydrolase<br>**Chain:** A: **PDB Molecule:**serine hydrolase ccsp0084;<br>**PDBTitle:** crystal structure of serine hydrolase ccsp0084 from the polyaromatic2 hydrocarbon (pah)-degrading bacterium cycloclasticus zankles<br>**PDB Entry:** PDBe RCSB PDBj
101	c6cl4A_	Alignment	not modelled	99.9	18	**PDB header:**hydrolase<br>**Chain:** A: **PDB Molecule:**lipase c12;<br>**PDBTitle:** lipc12 - lipase from metagenomics<br>**PDB Entry:** PDBe RCSB PDBj
102	c3fobA_	Alignment	not modelled	100.0	16	**PDB header:**oxidoreductase<br>**Chain:** A: **PDB Molecule:**bromoperoxidase;<br>**PDBTitle:** crystal structure of bromoperoxidase from bacillus anthracis<br>**PDB Entry:** PDBe RCSB PDBj
103	c5hdfB_	Alignment	not modelled	100.0	16	**PDB header:**hydrolase<br>**Chain:** B: **PDB Molecule:**hydrolase;<br>**PDBTitle:** hydrolase semet-stna<br>**PDB Entry:** PDBe RCSB PDBj
104	d2pl5a1	Alignment	not modelled	100.0	14	**Fold:** alpha/beta-Hydrolases<br>**Superfamily:** alpha/beta-Hydrolases<br>**Family:** O-acetyltransferase<br>**PDB entry:** PDBe RCSB PDBj
105	c5efzC_	Alignment	not modelled	100.0	14	**PDB header:**hydrolase<br>**Chain:** C: **PDB Molecule:**homoserine o-acetyltransferase;<br>**PDBTitle:** monoclinic structure of the acetyl esterase mekb<br>**PDB Entry:** PDBe RCSB PDBj
106	c4qlaB_	Alignment	not modelled	100.0	16	**PDB header:**hydrolase<br>**Chain:** B: **PDB Molecule:**juvenile hormone epoxide hydrolase;<br>**PDBTitle:** crystal structure of juvenile hormone epoxide hydrolase from the2 silkworm bombyx mori<br>**PDB Entry:** PDBe RCSB PDBj
107	c6qe2A_	Alignment	not modelled	100.0	16	**PDB header:**hydrolase<br>**Chain:** A: **PDB Molecule:**monoacylglycerol lipase;<br>**PDBTitle:** crystal structure of paleococcus ferrophilus monoacylglycerol lipase.<br>**PDB Entry:** PDBe RCSB PDBj
108	c4nvrC_	Alignment	not modelled	100.0	15	**PDB header:**transferase<br>**Chain:** C: **PDB Molecule:**putative acyltransferase;<br>**PDBTitle:** 2.22 angstrom resolution crystal structure of a putative2 acyltransferase from salmonella enterica<br>**PDB Entry:** PDBe RCSB PDBj
109	c3hjuB_	Alignment	not modelled	100.0	15	**PDB header:**hydrolase<br>**Chain:** B: **PDB Molecule:**monoglyceride lipase;<br>**PDBTitle:** crystal structure of human monoglyceride lipase<br>**PDB Entry:** PDBe RCSB PDBj
110	c3wzlB_	Alignment	not modelled	100.0	16	**PDB header:**hydrolase<br>**Chain:** B: **PDB Molecule:**zearalenone hydrolase;<br>**PDBTitle:** zen lactonase<br>**PDB Entry:** PDBe RCSB PDBj
111	c6azdA_	Alignment	not modelled	100.0	16	**PDB header:**hydrolase<br>**Chain:** A: **PDB Molecule:**ppkai2-like h;<br>**PDBTitle:** crystal structure of physcomitrella patens kai2-like h<br>**PDB Entry:** PDBe RCSB PDBj
112	d1va4a_	Alignment	not modelled	100.0	16	**Fold:** alpha/beta-Hydrolases<br>**Superfamily:** alpha/beta-Hydrolases<br>**Family:** Haloperoxidase<br>**PDB entry:** PDBe RCSB PDBj
113	c7e04A_	Alignment	not modelled	100.0	17	**PDB header:**hydrolase<br>**Chain:** A: **PDB Molecule:**lipase;<br>**PDBTitle:** crystal structure of bomgl, a monoacylglycerol lipase from marine2 bacillus sp.<br>**PDB Entry:** PDBe RCSB PDBj
114	c4q3kB_	Alignment	not modelled	99.2	19	**PDB header:**hydrolase<br>**Chain:** B: **PDB Molecule:**mgs-m1;<br>**PDBTitle:** crystal structure of mgs-m1, an alpha/beta hydrolase enzyme from a2 medee basin deep-sea metagenome library<br>**PDB Entry:** PDBe RCSB PDBj
115	c5oluA_	Alignment	not modelled	100.0	15	**PDB header:**hydrolase<br>**Chain:** A: **PDB Molecule:**alpha/beta hydrolase family protein;<br>**PDBTitle:** the crystal structure of a highly thermostable carboxyl

						esterase from2 bacillus coagulans in complex with glycerol<br>**PDB Entry:** PDBe RCSB PDBj
116	c5jd6A_	Alignment	not modelled	100.0	19	**PDB header:**hydrolase<br>**Chain:** A: **PDB Molecule:**mgs-mche2;<br>**PDBTitle:** crystal structure of mgs-mche2, an alpha/beta hydrolase enzyme from2 the metagenome of sediments from the lagoon of mar chica, morocco<br>**PDB Entry:** PDBe RCSB PDBj
117	c4f0jA_	Alignment	not modelled	100.0	14	**PDB header:**hydrolase<br>**Chain:** A: **PDB Molecule:**probable hydrolytic enzyme;<br>**PDBTitle:** crystal structure of a probable hydrolytic enzyme (pa3053) from2 pseudomonas aeruginosa pao1 at 1.50 a resolution<br>**PDB Entry:** PDBe RCSB PDBj
118	d1imja_	Alignment	not modelled	100.0	21	**Fold:** alpha/beta-Hydrolases<br>**Superfamily:** alpha/beta-Hydrolases<br>**Family:** Ccg1/TafII250-interacting factor B (Cib)<br>**PDB entry:** PDBe RCSB PDBj
119	c7a6gA_	Alignment	not modelled	100.0	15	**PDB header:**hydrolase<br>**Chain:** A: **PDB Molecule:**putative proline iminopeptidase;<br>**PDBTitle:** structural characterization of l-proline amide hydrolase from2 pseudomonas syringae<br>**PDB Entry:** PDBe RCSB PDBj
120	c6avyA_	Alignment	not modelled	99.9	18	**PDB header:**hydrolase<br>**Chain:** A: **PDB Molecule:**acyl-protein thioesterase 2;<br>**PDBTitle:** crystal structure of zea mays acyl-protein thioesterase 2<br>**PDB Entry:** PDBe RCSB PDBj
